# Racial and Ethnic Disparities in Non-Fatal Self-Inflicted Firearm Injuries Among Adults in the United States, 2000–2021

**DOI:** 10.1007/s10900-026-01592-9

**Published:** 2026-06-17

**Authors:** Gia Elise Barboza-Salerno, Malcolm McCarthy, Amy Watson-Grace, Taylor Harrington, Bruna De AtalayaAlmieda Rocha, Zarah Alhajjaj, Keith Warren, Karla Shockley McCarthy

**Affiliations:** 1 College of Public Health, The Ohio State University, Columbus, OH USA; 2 College of Social Work, The Ohio State University, Columbus, OH USA; 3 Department of Psychology, The Ohio State University, Columbus, OH USA; 4 School of Health and Rehabilitation Sciences, The Ohio State University, Columbus, OH USA; 5 College of Nursing, The Ohio State University, Columbus, OH USA; 6 Ohio Colleges of Medicine Government Resource Center, Columbus, OH USA

**Keywords:** Firearms, Self-inflicted, Gunshot wound, Natural Language Processing, Suicide, Self-harm, Unintentional, Race/ethnicity, NEISS-FISS

## Abstract

Nonfatal self-inflicted firearm injuries represent an important and understudied public health concern in the United States. We analyzed nationally representative data from the National Electronic Injury Surveillance System–Firearm Injury Surveillance Study (NEISS-FISS), 2000–2021, to characterize nonfatal self-inflicted firearm injuries among U.S. adults aged 18–64 years. Emergency department (ED) narratives were combined using text mining and an elastic net (LASSO) classification model to augment structured surveillance data. Among 7,362 unweighted cases (representing approximately 260,602 ED visits, 58% were classified as probable unintentional discharges, whereas approximately 10% were probable suicide attempts. Among cases with complete race and ethnicity data, Non-Hispanic White (NHW; 69.2%), Non-Hispanic Black (NHB; 23.2%), and Hispanic (7.6%) individuals comprised the primary analytic groups. Narrative analysis substantially increased detection of contextual factors, particularly alcohol involvement among Hispanic patients (12.0% to 20.1%). NHB patients had the highest proportion of lower-extremity injuries (51%), while Hispanic patients had the highest prevalence of craniofacial injuries (26%). The LASSO model effectively distinguished terms associated with probable suicide attempts from unintentional firearm discharges based on narrative language. Findings highlight limitations of structured intent coding within NEISS-FISS and demonstrate the value of narrative for improving characterization of firearm injury circumstances, revealing distinct racial and ethnic group patterns, to inform more tailored prevention strategies.

**Supplementary Information:**

The online version contains supplementary material available at https://doi.org/10.1007/s10900-026-01592-9.

## Background

Nonfatal self-inflicted firearm injury is a significant and ongoing public health concern in the United States [1]. Although self-inflicted firearm injuries have an exceptionally high fatality rate (89.4%-90.9%), thousands of individuals survive each year, with recent estimates ranging from 2,501 to 3,293 nonfatal cases annually [1, 4]. While these cases represent a small proportion of all nonfatal firearm injuries (2.9%-4%), they contribute substantially to the healthcare burden, with total annual costs rising from $0.59 billion in 2019 to $0.67 billion in 2020, and overall firearm injury hospital costs exceeding $1 billion annually in the U.S [1–6]. Additionally, survivors of a self-inflicted firearm injury experience substantial short- and long-term consequences, including: increased pain and anxiety, acute reduced physical functioning, chronic pain, long-term functional limitations, poorer mental and physical health, lower employment and return-to-work rates, diminished social functioning, and elevated substance use [2, 3]. Despite this substantial burden, national evidence on nonfatal self-inflicted firearm injury remains limited. Prior research has often relied on single-center retrospective chart reviews with small sample sizes or has narrowly focused on acute clinical outcomes [7]. As a result, there is a lack of comprehensive, nationally representative analyses that characterize the epidemiology, injury patterns, and broader context of these events over time.

A major challenge in documenting racial and ethnic disparities in firearm injuries across the U.S. is the lack of nationally representative data. While disparities in fatalities are well known, less is understood about nonfatal firearm injuries, especially self-inflicted ones [4, 8, 9]. Evidence indicates that minoritized groups, particularly Non-Hispanic Black (NHB) Americans, are disproportionately affected by nonfatal firearm injuries, accounting for a large share of emergency department (ED) visits and experiencing significantly higher rates of firearm assault-related injuries [4, 10]. Although they constitute only 12.6% of the population, NHB individuals account for 61.5% of firearm assaults and have nonfatal assault rates twenty times those of Non-Hispanic White (NHW) individuals, with an outsized impact on NHB males aged 15 to 34 [4]. Additionally, trauma registry data demonstrate that NHB patients are the only racial group with a consistent increase in firearm-related hospitalizations over time [11]. These disparities are evident across injury contexts: NHB individuals represent 31.6% of civilian injuries from law enforcement firearm activity and face the highest rates of nonfatal unintentional firearm injuries [4, 12]. Conversely, NHW individuals experience the highest rates of nonfatal self-harm injuries, while Native American populations have the highest case-fatality ratios for firearm assaults and law enforcement-related injuries [4]. Overall, these patterns demonstrate how race and ethnicity intersect with intent, exposure, and injury severity, underscoring the need for further understanding of how these factors influence disparities in nonfatal firearm injuries.

A persistent barrier to advancing understanding of the nature and circumstances surrounding self-inflicted harm is the limited ability of administrative data to accurately capture injury intent and additional contextual factors. Many surveillance systems rely heavily on structured coding fields that may omit important details or misclassify the circumstances surrounding an injury event. For example, national databases such as the Nationwide Emergency Department Sample (NEDS) have only recently improved the collection and reporting of race and ethnicity data, limiting earlier studies’ ability to examine disparities with precision. Prior research has demonstrated that text narratives can improve intent classification, reduce missingness through data imputation, and support the application of natural language processing and machine learning approaches to better characterize firearm injury events and their surrounding contexts [13, 14]. Using the National Electronic Injury Surveillance System Firearm Injury Surveillance Study (NEISS-FISS) provides an important opportunity to address several of these limitations. In addition to structured variables, NEISS-FISS contains detailed free-text ED narratives describing the circumstances of firearm injuries. These narratives, generated through manual abstraction by trained coders, provide contextual information regarding injury mechanisms, shooter identity, and situational characteristics that are often unavailable or inaccurately represented in structured fields [13, 15]. Prior research has demonstrated that these narratives can improve intent classification, reduce missingness through data imputation, and support the application of natural language processing and machine learning approaches to better encapsulate the firearm injury events and their surrounding contexts [13].

Building on this foundation, the present study integrates nationally representative surveillance data with narrative text analysis to address key gaps in the literature. Specifically, this study has three aims: (1) to examine racial and ethnic differences in temporal patterns and epidemiology of nonfatal self-inflicted firearm injuries using segmented Joinpoint regression; (2) to evaluate the utility of narrative text to improve the classification of firearm injury intent across ethnoracial groups; (3) to characterize racial and ethnic differences in injury mechanisms and contextual circumstances using narrative text mining and machine learning approaches, distinguishing probable suicide attempts from unintentional discharges.

### Data

We conducted a retrospective cross-sectional analysis limited to self-inflicted non-fatal firearm–related ED visits in the United States from 2000 to 2021. Data were obtained from the National Electronic Injury Surveillance System Firearm Injury Surveillance Study (NEISS-FISS), maintained by the U.S. Consumer Product Safety Commission (CPSC) in collaboration with the Centers for Disease Control and Prevention (CDC). NEISS-FISS is a nationally representative, stratified probability sample of approximately 100 U.S. hospitals with at least six inpatient beds and 24-hour emergency services. The system systematically captures ED presentations in which a firearm or gun is documented in the medical record, including penetrating gunshot injuries, non-penetrating firearm-related injuries, and other firearm-associated incidents across participating hospital strata. The survey employs a complex sampling design with stratification by hospital size and geographic region, and provides case-level sampling weights to generate nationally representative estimates. Data for the present study were drawn from the 1993–2021 public-use release available through the Inter-university Consortium for Political and Social Research [16]. Although the full dataset spans 1993 onward, analyses were restricted to cases occurring between 2000 and 2021. Ethical review was not required because all data are publicly available and fully de-identified.

The analytic sample was constructed using a series of inclusion criteria. First, cases were restricted to confirmed nonfatal firearm injuries, including only penetrating projectile injuries and excluding non-penetrating firearm-related incidents such as struck-by events. Second, injuries were required to be coded as self-inflicted. Within NEISS-FISS, self-inflicted injuries are defined as incidents in which the injured person also discharged the firearm; however, this classification broadly encompasses both intentional and unintentional mechanisms. Third, the analytic sample was restricted to adults aged 18 to 64 years. Finally, analyses were limited to incidents occurring between 2000 and 2021.

Application of these criteria yielded an unweighted analytic sample of 7,362 cases, corresponding to a weighted national estimate of 260,602 ED visits (SE = 54,268) during the study period. Primary stratified analyses focused on NHW, NHB, and Hispanic patients, who together accounted for 5,572 cases with complete race and ethnicity information. Cases identified as Asian, American Indian/Alaska Native, or Other race were retained in the overall analyses but excluded from the race-stratified analyses due to small cell sizes and concerns about the stability of estimates.

### Variables

Race and ethnicity were derived from separate race and Hispanic ethnicity fields and combined into a single classification variable: NHW, NHB, Hispanic, Asian, American Indian/Alaska Native, and Other. Patient age was categorized as 18–25, 26–45, and 46–64 years. Five-year analytic periods (2000–2004 through 2020–2021) were created for temporal trend analyses. The primary body-part code was collapsed into five anatomical injury regions: craniofacial/head-neck, trunk and internal/body systems, upper extremity, lower extremity, and multiple or all body regions. ED disposition was classified as treated and released, transferred, hospitalized, observed, or left against medical advice. A binary transport variable indicated arrival by emergency medical services or ambulance versus all other transport modes.

Annual injury rates per 100,000 person-years were calculated separately for each racial and ethnic group using race-specific population denominators. Following Bhagavathula et al. [17], for 2000–2019, denominators were obtained from CDC WONDER (Wide-ranging Online Data for Epidemiologic Research) Bridged-Race Population Estimates; for 2020–2021, denominators were obtained from Census Bureau Single-Race Population Estimates. Population files were restricted to individuals aged 18–64 years and aggregated by state, single-year age category, racial and ethnic group, and calendar year, before being merged with weighted injury counts.

The ED narrative text field was complete for all 7,362 analytic cases and was used to supplement and recover information from structured variables that were frequently coded as unknown or insufficiently specified. Binary indicators derived from narrative text were developed to identify probable suicide attempts, probable unintentional firearm discharges, law-enforcement-related contexts, substance involvement, physical fight context, crime involvement, verbal argument context, firearm type, and incident location.

### Analysis

All analyses were conducted in R. The complex survey design incorporated primary sampling units, strata, and case-level sampling weights to account for clustering within strata. Weighted descriptive statistics are presented as unweighted counts and weighted percentages, reported as *n (%)*. Group differences were evaluated using Rao–Scott chi-square tests, which extend Pearson chi-square procedures for complex survey data. Statistical significance was assessed using two-sided tests with α = 0.05. Missing values in structured categorical variables were retained as separate categories or, where appropriate, coded as unknown to preserve analytic sample size and to evaluate information recovered through narrative augmentation.

Narrative text processing followed a multistage preprocessing pipeline prior to tokenization. Narrative fields were cleaned through contraction expansion, URL removal, abbreviation standardization, misspelling correction, and removal of punctuation and numeric characters. Text was subsequently lemmatized using the *textstem* package [18] and tokenized into individual terms. Standard English stop words and a customized domain-specific stop-word list were removed. Remaining terms underwent synonym harmonization procedures that collapsed clinically and semantically similar expressions into common canonical forms, reducing sparsity and improving interpretability. Additional filtering removed infrequent terms, noninformative tokens, and predefined terms that could be associated with label leakage in downstream prediction models.

Narrative-derived indicators were developed using rule-based text classification procedures to identify probable suicide attempts, unintentional firearm discharges, law-enforcement-related contexts, substance involvement, and other contextual characteristics. Structured intent classifications were cross-tabulated against text-derived classifications to identify discordant cases. Cases containing explicit suicidal language, but conflicting structured intent classifications, were flagged for further review and sensitivity assessment.

To identify terms disproportionately represented across racial and ethnic groups, log-odds ratios with Laplace smoothing were calculated for terms appearing in at least 10 cases. These analyses compared the odds of a term occurring within a specific racial or ethnic group relative to the remaining groups combined. The top distinguishing terms for each group were retained for interpretation and visualization. Temporal trends in annual injury rates per 100,000 population were evaluated using segmented joinpoint regression models fit separately by racial and ethnic group. Models were applied to log-transformed annual rates and specified a single changepoint, producing two piecewise linear segments and an estimated transition year. Annual percent changes and confidence intervals were derived from model coefficients using the delta method.

Finally, an elastic net penalized logistic regression model with a mixing parameter of 0.8 was developed to distinguish probable suicide attempts from probable unintentional firearm discharges using sparse document-term matrices generated from narrative text and structured patient characteristics. To minimize temporal information leakage, a strict temporal split was applied, with cases from 2000 to 2010 used for model training and cases from 2011 to 2021 reserved for out-of-sample testing. Vocabulary construction was restricted to the training data and subsequently applied to the test set. Model tuning used 10-fold cross-validation during the training period. Predictive performance was evaluated using accuracy, sensitivity, specificity, positive predictive value, negative predictive value, F1 score, and area under the receiver operating characteristic curve.

## Results

### Sample Characteristics

Between 2000 and 2021, 7,362 unweighted cases of non-fatal self-inflicted firearm injuries in adults aged 18–64 years were identified in the NEISS-FISS, representing an estimated 260,602 (SE = 54,268) nationally representative ED visits. Table 1 presents the full sociodemographic and clinical profile stratified by race/ethnicity. Of the 5,572 cases with complete race/ethnicity data, 3,858 (69.2%) were NHW, 1,293 (23.2%) were NHB, and 421 (7.6%) were Hispanic.

**Table 1:** Nonfatal self-inflicted firearm gunshot wounds by race/ethnicity, United States, 2000–2021 (unweighted n, weighted %)

Characteristic	Overall *N* = 5,572	Non-Hispanic White *N* = 3,858	Non-Hispanic Black *N* = 1,293	Hispanic *N* = 421
Sex (*p* = 0.051)				
Male	4,887 (88%)	3,338 (88%)	1,159 (89%)	390 (92%)
Female	684 (12%)	519 (12%)	134 (11%)	31 (8.2%)
Age Group (*p* < 0.001)				
18–25 years	1,735 (31%)	1,015 (27%)	535 (43%)	185 (45%)
26–45 years	2,418 (43%)	1,669 (43%)	567 (43%)	182 (42%)
46–64 years	1,419 (26%)	1,174 (30%)	191 (15%)	54 (13%)
Intent (from narrative) (*p* < 0.001)				
Probable suicide attempt	604 (10%)	484 (11%)	75 (5.1%)	45 (12%)
Probable unintentional	3,167 (58%)	2,065 (56%)	845 (65%)	257 (61%)
Self-harm (no text signal)	738 (15%)	520 (16%)	185 (16%)	33 (7.6%)
Law enforcement context	35 (0.6%)	19 (0.5%)	10 (1.0%)	6 (1.0%)
Indeterminate	1,028 (16%)	770 (16%)	178 (13%)	80 (18%)
Firearm Type				
Handgun	3,040 (54%)	2,114 (55%)	703 (53%)	223 (53%)
Long gun (rifle/shotgun)	655 (14%)	558 (16%)	58 (5.4%)	39 (8.1%)
Unknown	1,876 (32%)	1,185 (29%)	532 (42%)	159 (39%)
Alcohol/Substance Use				
Yes	499 (10%)	316 (9.4%)	110 (8.4%)	73 (20%)
No	1,594 (30%)	1,065 (29%)	406 (34%)	123 (29%)
Unknown	3,479 (60%)	2,477 (62%)	777 (57%)	225 (51%)
Physical Fight (*p* = 0.6)				
Yes	132 (2.1%)	89 (2.0%)	26 (1.8%)	17 (3.1%)
No	1,197 (23%)	830 (22%)	261 (23%)	106 (26%)
Unknown	4,243 (75%)	2,939 (76%)	1,006 (75%)	298 (71%)
Crime Involvement (*p* = 0.055)				
Yes	72 (1.2%)	40 (0.8%)	18 (2.0%)	14 (3.5%)
No	1,252 (23%)	838 (22%)	315 (27%)	99 (23%)
Unknown	4,248 (76%)	2,980 (77%)	960 (71%)	308 (73%)
Verbal Argument (*p* = 0.4)				
Yes	130 (2.3%)	96 (2.4%)	21 (1.8%)	13 (3.2%)
No	1,294 (24%)	899 (24%)	277 (23%)	118 (29%)
Unknown	4,148 (73%)	2,863 (74%)	995 (75%)	290 (68%)
Location (*p* = 0.007)				
Home/residence	2,418 (43%)	1,564 (42%)	683 (54%)	171 (35%)
Public/transport	85 (2.0%)	68 (2.2%)	10 (1.1%)	7 (2.2%)
Other known	486 (9.0%)	306 (8.2%)	132 (10%)	48 (13%)
Unknown	2,583 (46%)	1,920 (48%)	468 (35%)	195 (50%)
ED Disposition (*p* < 0.001)				
Treated	2,396 (46%)	1,529 (44%)	693 (57%)	174 (41%)
Hospitalized	2,751 (43%)	2,001 (43%)	534 (35%)	216 (53%)
Transferred	316 (9.2%)	254 (10%)	50 (6.4%)	12 (3.2%)
Observed	79 (1.6%)	53 (1.6%)	8 (0.7%)	18 (3.3%)
AMA/LWBS	30 (0.7%)	21 (0.7%)	8 (0.8%)	1 (< 0.1%)
Injury Region (*p* < 0.001)				
Lower extremity	2,050 (38%)	1,263 (35%)	635 (51%)	152 (37%)
Upper extremity	1,152 (21%)	811 (22%)	244 (18%)	97 (21%)
Craniofacial/head-neck	1,143 (19%)	885 (20%)	155 (11%)	103 (26%)
Trunk/internal	835 (14%)	638 (15%)	147 (10%)	50 (11%)
Multiple/all body	384 (7.9%)	256 (7.8%)	109 (9.8%)	19 (5.1%)
Transported by EMS (*p* = 0.4)				
Yes	1,023 (15%)	703 (15%)	259 (17%)	61 (15%)
No	4,549 (85%)	3,155 (85%)	1,034 (83%)	360 (85%)

NHW = Non-Hispanic White; NHB = Non-Hispanic Black. p-values based on Rao-Scott chi-square test. n (%) = unweighted count (weighted percent). AMA = against medical advice; LWBS = left without being seen. Intent classification is text-derived from the narrative field (see Methods). Firearm type, substance use, fight, crime, argument, and location are text-resolved (see Methods and Table 2)

Males constituted 88% of cases overall, with no statistically significant difference by race/ethnicity (*p* = 0.051). Age distributions differed significantly (*p* < 0.001): NHW patients were substantially older, with 30% aged 46–64 years, compared with 15% of NHB patients and 13% of Hispanic patients. Conversely, 43–45% of NHB and Hispanic patients were in the youngest age group (18–25 years), compared with 27% of NHW patients.

### Injury Rate Trends and Joinpoint Analysis

**Fig. 1:**
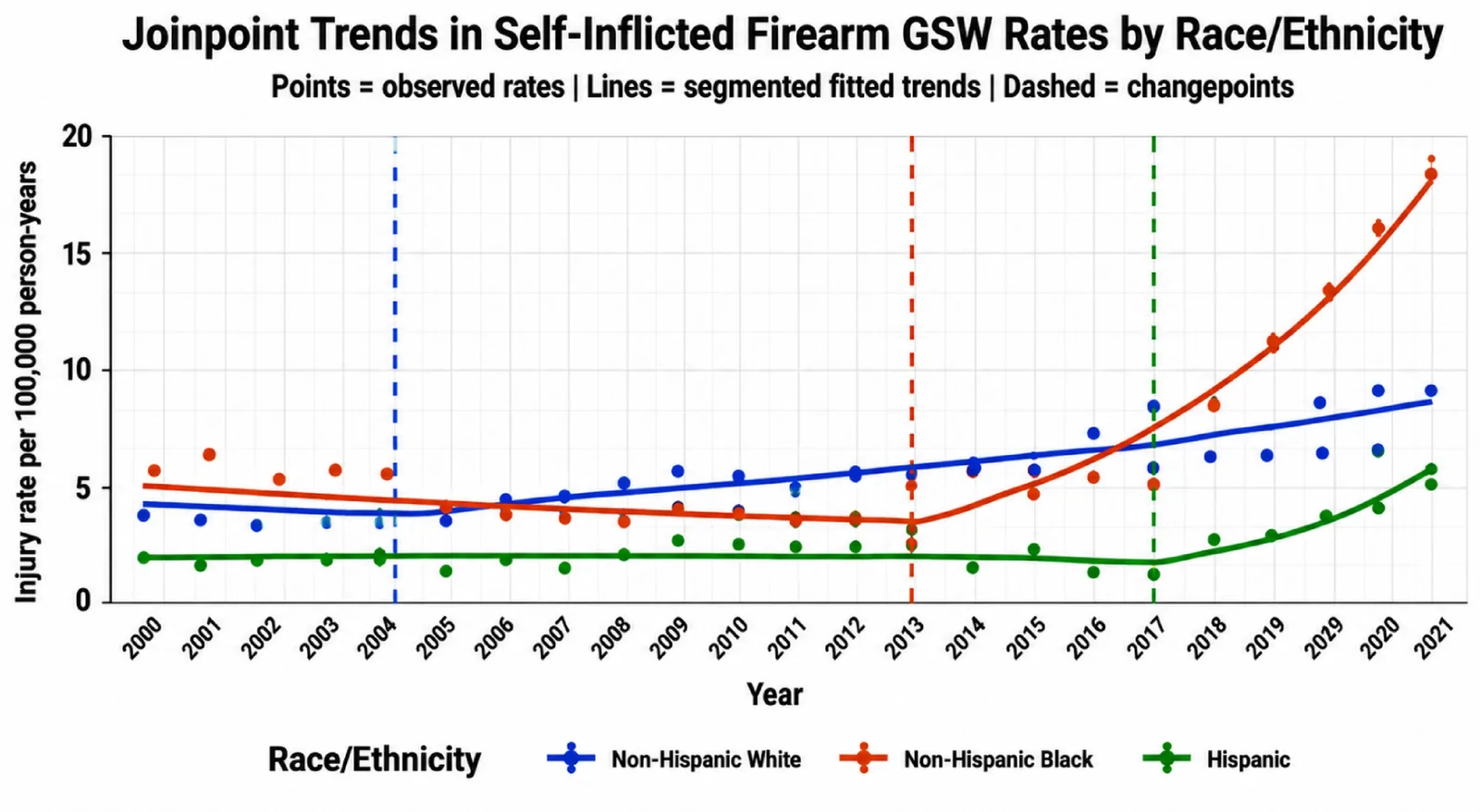
Age-Standardized Incidence of nonfatal firearm injury by race/ethnicity in the USA from 2000–2021. Joinpoint regression analysis indicated that the best-fitting model includes two distinct trends (one joinpoint) for each racial/ethnic group. For the Non-Hispanic White population, the estimated changepoint occurred in 2004 (SE = 1.51), with a non-significant decline prior to the changepoint (APC = − 3.8%, 95% CI: −10.9 to 3.9, *p* = 0.327) followed by a significant increase of 4.4% annually after 2004 (95% CI: 3.2–5.7, *p* < 0.001). For the Non-Hispanic Black population, the changepoint occurred in 2013 (SE = 0.79), with a significant decline prior to 2013 (APC = − 3.2%, 95% CI: −6.2 to − 0.2, *p* = 0.037) followed by a sharp and significant increase of 21.7% annually after 2013 (95% CI: 15.4–28.5, *p* < 0.001). For the Hispanic population, the changepoint occurred in 2017 (SE = 1.28), with a non-significant pre-changepoint trend (APC = − 1.8%, *p* = 0.249) followed by a steep increase of 39.6% annually after 2017 (95% CI: 3.2–88.9, *p* = 0.030). By 2021, Non-Hispanic Black individuals had the highest injury rates (17.2 per 100,000 person-years), substantially exceeding rates among Non-Hispanic White (8.5) and Hispanic (5.3) populations. A pronounced V-shaped trajectory was observed among Non-Hispanic Black individuals, with declining rates from 2000 to 2013, followed by rapid increases thereafter. Points represent observed annual rates, solid lines represent segmented fitted trends, and dashed vertical lines indicate estimated changepoints

Annual injury rates per 100,000 person-years increased across all three groups over the study period, with marked acceleration beginning in the mid-2010s (Fig. 1; Table 2). NHW patients had the earliest changepoint (estimated year 2004; SE = 1.51), with a non-significant pre-changepoint decline of 3.8% annually (95% CI: −10.9 to + 3.9%; *p* = 0.327) followed by a significant increase of 4.4% annually after 2004 (95% CI: 3.2–5.7%; *p* < 0.001). NHB patients had a changepoint at 2013 (SE = 0.79), with a significant pre-changepoint decline of 3.2% annually (95% CI: −6.2 to − 0.2%; *p* = 0.037) followed by a dramatic acceleration of 21.7% annually after 2013 (95% CI: 15.4–28.5%; *p* < 0.001). Hispanic patients had the latest changepoint (2017; SE = 1.28), with a non-significant pre-changepoint decline of 1.8% annually (*p* = 0.249) followed by an increase of 39.6% annually after 2017 (95% CI: 3.2–88.9%; *p* = 0.030). By 2021, NHB patients had reached 17.2 per 100,000 person-years, far exceeding NHW (8.5) and Hispanic (5.3) rates. A notable V-shaped trajectory was observed for NHB patients, whose rates declined through 2013 before reversing sharply, a pattern not explained by population change. Figure 1 presents the segmented joinpoint trend fits with estimated changepoints.

**Table 2:** Annual percent change (APC) in self-inflicted firearm GSW rates by race/ethnicity, 2000–2021, from joinpoint (segmented) regression on log-transformed annual rates per 100,000 person-years

Race/Ethnicity	Segment	β	SE	z	*p*-value	APC (%)	95% CI
Non-Hispanic White	Pre-changepoint (2000–2004)	-0.038	0.039	-0.98	0.327	-3.8	-10.9 to + 3.9
Non-Hispanic White	Post-changepoint (2004–2021)	0.043	0.006	7.04	< 0.001	+ 4.4	3.2 to 5.7
Non-Hispanic Black	Pre-changepoint (2000–2013)	-0.033	0.016	-2.08	0.037	-3.2	-6.2 to -0.2
Non-Hispanic Black	Post-changepoint (2013–2021)	0.197	0.027	7.17	< 0.001	+ 21.7	15.4 to 28.5
Hispanic	Pre-changepoint (2000–2017)	-0.018	0.016	-1.15	0.249	-1.8	-4.8 to + 1.3
Hispanic	Post-changepoint (2017–2021)	0.334	0.154	2.17	0.030	+ 39.6	3.2 to 88.9

APC = annual percent change; CI = 95% confidence interval. NHW = Non-Hispanic White; NHB = Non-Hispanic Black; Changepoint years: NHW = 2004 (SE 1.51); NHB = 2013 (SE 0.79); Hispanic = 2017 (SE 1.28). Model fit by segmented regression on log(rate); k = 1 joinpoint per group

### Temporal Variation in Clinical Characteristics

Table 3 presents temporal trends in injury characteristics across five periods from 2000 to 2004 through 2020–2021, stratified by race/ethnicity. The supplemental temporal figures (Figures S1–S6) illustrate these trends. Several patterns are worth noting. Among NHB patients, the proportion of lower extremity injuries increased dramatically from 36% in 2000–2004 to 60% in 2020–2021 (+ 68%), while craniofacial injuries declined from 12% to 10%, a shift not observed among NHW or Hispanic patients. The proportion of cases with unknown firearm type increased substantially across all groups, reaching 35% for NHW, 49% for NHB, and 52% for Hispanic patients in 2020–2021. Among Hispanic patients, the 26–45-year age group increased from 34% to 53% (+ 19%) of cases, while the youngest group (18–25 years) declined. Substance involvement remained highly variable and low across all groups, reflecting the high rate of unknown coding rather than true absence.

**Table 3:** Temporal variation in selected characteristics of non-fatal self-inflicted firearm GSW by race/ethnicity and five-year period, 2000–2021 (weighted proportions, %)

Variable	Category	Race/Ethnicity	2000–2004	2005–2009	2010–2014	2015–2019	2020–2021	% change
Age group	18–25 years	NHW	24.9	28.5	27.4	25.5	28.7	+ 15.2
		NHB	50.5	42.0	48.9	39.1	38.2	-24.4
		Hispanic	54.8	45.2	48.1	44.3	37.1	-32.2
	26–45 years	NHW	47.2	44.6	41.1	40.3	46.2	-2.1
		NHB	30.7	45.3	36.6	46.1	48.6	+ 58.2
		Hispanic	34.0	34.8	44.8	36.5	53.4	+ 57.0
Injury region	Lower extremity	NHW	31.0	34.3	37.3	34.9	36.7	+ 18.4
		NHB	35.9	48.6	42.6	57.0	60.2	+ 67.9
		Hispanic	39.3	32.9	30.9	41.9	41.0	+ 4.2
	Craniofacial/head-neck	NHW	20.2	17.7	19.9	22.1	22.1	+ 9.4
		NHB	12.4	11.3	12.7	9.8	9.5	-23.5
		Hispanic	28.2	24.6	19.5	27.0	29.3	+ 3.9
Firearm type	Handgun	NHW	54.4	56.1	54.8	54.5	54.8	+ 0.7
		NHB	50.6	63.1	64.1	46.8	47.5	-6.2
		Hispanic	58.2	55.6	54.9	52.3	44.2	-24.0
	Unknown	NHW	21.4	26.7	28.3	33.0	35.2	+ 64.4
		NHB	40.1	30.3	31.9	50.0	49.0	+ 22.4
		Hispanic	27.4	33.3	35.7	42.6	52.4	+ 91.4
Alcohol/substance	Yes	NHW	7.1	2.3	3.0	4.4	6.9	-2.8
		NHB	3.1	3.2	2.1	6.4	5.8	+ 85.8
		Hispanic	17.8	8.2	10.5	8.5	15.8	-11.3
ED disposition	Hospitalized	NHW	47.5	41.9	43.2	42.5	41.5	-12.6
		NHB	34.6	32.9	30.4	36.5	35.8	+ 3.6
		Hispanic	58.1	44.1	52.4	62.3	47.1	-18.9
	Treated	NHW	40.5	45.5	42.7	45.0	45.9	+ 13.2
		NHB	57.0	57.4	64.2	55.3	56.5	-1.0
		Hispanic	39.5	53.0	36.0	35.5	41.7	+ 5.6
Transported by EMS	Yes	NHW	20.6	12.7	11.8	12.2	22.1	+ 7.4
		NHB	20.8	14.4	10.1	13.5	25.2	+ 20.9
		Hispanic	36.8	16.2	7.3	8.6	12.3	-66.5

Values are weighted percentages. NHW = Non-Hispanic White; NHB = Non-Hispanic Black. % Δ = percent change from 2000–2004 to 2020–2021 period. Selected rows shown; complete data available in supplemental material. Alcohol/substance reflects structured coding

### Agreement and Discordance between Structured and Narrative Data

The text-derived intent classification revealed that the majority of self-inflicted injury cases were probable unintentional/accidental discharges (58% overall), not suicide attempts. Probable suicide attempts accounted for only 10% of cases, though this proportion differed significantly by race/ethnicity (*p* < 0.001): 11% among NHW, 5.1% among NHB, and 12% among Hispanic patients. NHB patients had the highest proportion of probable unintentional cases (65%), exceeding NHW (56%) and Hispanic (61%) patients. The category “self-harm (no text signal)” represents cases coded as self-harm in the structured data but without corroborating suicidal language in the narrative, comprised 15–16% of cases in NHW and NHB patients, but only 7.6% in Hispanic patients.

During systematic data validation, 754 cases were identified in which the structured NEISS-FISS intent code indicated law enforcement involvement. Of these, 741 cases (36.1% of all law-enforcement-coded cases) contained explicit suicidal language within the ED narrative but no corresponding law enforcement terminology, suggesting potential discordance between structured coding and narrative content. An additional 13 cases contained both suicide-related and law enforcement language, indicating possible police-context events that could not be definitively reclassified. These discordant cases spanned the full 22-year study period and all three primary racial and ethnic groups.

Independent manual review of a subset of sample narratives confirmed the nature of this discordance. Representative narratives from this group are shown in Appendix A, Supplementary Table 1. These cases often described a private, self-inflicted suicide attempt; none documented police or law enforcement involvement in causing the injury. These cases were recoded as probable suicide attempts based on narrative evidence.

Table 4 presents weighted proportions of structured versus text-resolved contextual variables with the highest frequency of cases coded as unknown or not enough information. Narrative augmentation produced the largest gains for alcohol and substance involvement across racial and ethnic groups. Among Hispanic patients, alcohol and substance involvement increased from 12.0% under structured coding alone to 20.1% following narrative augmentation (+ 8.1% points). Corresponding increases were observed among NHW patients (4.5% to 9.4%; +4.9% points) and NHB patients (4.6% to 8.4%; +3.8% points). Physical fight involvement showed smaller gains, ranging from + 0.6 to + 1.1% points across groups. In contrast, crime involvement and verbal argument variables demonstrated minimal or no change after narrative augmentation, suggesting these characteristics were already reasonably captured through structured surveillance coding. Overall, ED narratives primarily improved ascertainment of contextual and behavioral characteristics that appeared underrepresented in structured administrative fields.

**Table 4:** Structured versus text-resolved variable proportions by race/ethnicity, 2000–2021

Variable	Race/Ethnicity	% Yes (structured)	% Yes (text-resolved)	Change
Alcohol/substance use	Non-Hispanic White	4.5	9.4	+ 4.9
	Non-Hispanic Black	4.6	8.4	+ 3.8
	Hispanic	12.0	20.1	+ 8.1
Physical fight	Non-Hispanic White	1.0	2.0	+ 1.0
	Non-Hispanic Black	1.2	1.8	+ 0.6
	Hispanic	2.0	3.1	+ 1.1
Crime involvement	Non-Hispanic White	0.8	0.8	0.0
	Non-Hispanic Black	1.9	2.0	+ 0.1
	Hispanic	3.4	3.5	+ 0.1
Verbal argument	Non-Hispanic White	2.4	2.4	0.0
	Non-Hispanic Black	1.8	1.8	0.0
	Hispanic	3.2	3.2	0.0

Structured = proportions from coded NEISS-FISS variable. Text-resolved = proportions after applying keyword text mining to recover unknown-coded cases. Δ = text-resolved minus structured (percentage points). NHW = Non-Hispanic White; NHB = Non-Hispanic Black

### Clinical Characteristics and Injury Patterns

Firearm type did not differ significantly by race/ethnicity after text resolution (*p* = 0.4), with handguns the dominant identified type across all groups (~ 53–55%). Long gun (rifle/shotgun) involvement was substantially higher among NHW patients (16%) than NHB (5.4%) or Hispanic (8.1%) patients. Unknown firearm type remained high among NHB (42%) and Hispanic (39%) patients, limiting interpretation.

Injury region differed strongly by race/ethnicity (*p* < 0.001). Lower extremity injuries were the most common site among NHB patients (51%), substantially exceeding those among NHW (35%) and Hispanic (37%) patients. Hispanic patients had the highest rate of craniofacial/head-neck injuries (26%), compared to 20% for NHW and 11% for NHB, a pattern consistent with higher proportions of probable suicide attempt cases in those groups, given that craniofacial anatomy is disproportionately associated with intentional self-harm. NHW patients had the highest trunk/internal injury rate (15%). ED disposition differed significantly by race/ethnicity (*p* < 0.001). NHB patients were most frequently treated and released (57%), while NHW patients had the highest hospitalization rate (43%). Hispanic patients had the highest observed rate (3.3%) and the highest hospitalization rate (53%) among the three groups, consistent with their higher craniofacial injury prevalence, which is associated with greater clinical severity.

### Narrative Text Analysis

**Fig. 2:**
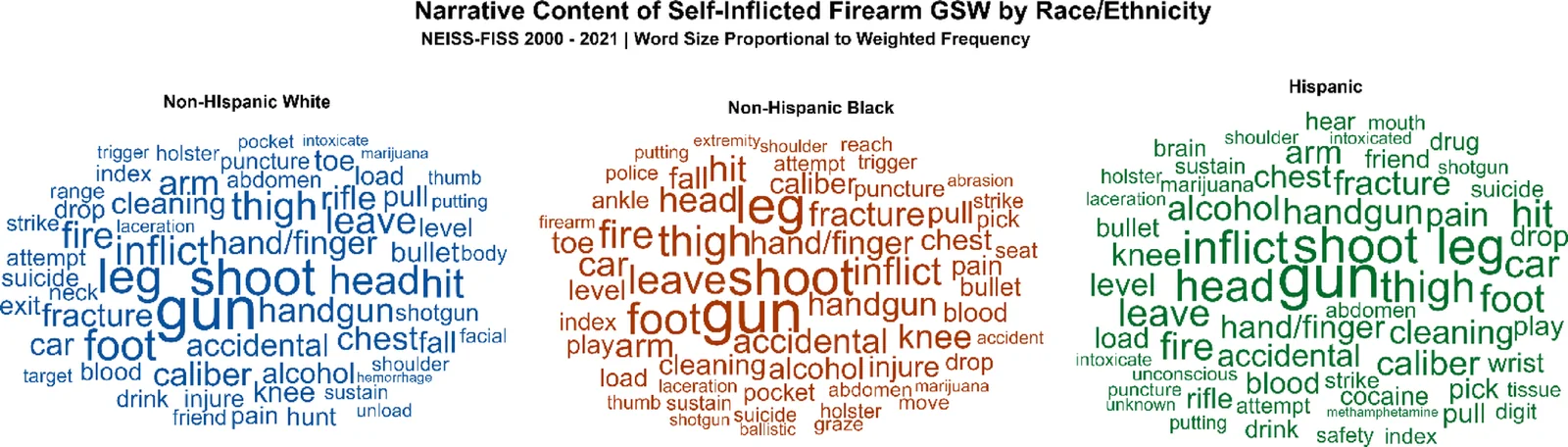
Narrative content of non-fatal self-inflicted firearm GSW by race/ethnicity, 2000–2021. Word size is proportional to weighted national frequency. Terms appearing in fewer than 5 cases are excluded. The top terms across all three groups were shoot, gun, and hand/finger, reflecting the predominance of unintentional handgun discharges

Figure 2 presents word clouds for each racial and ethnic group, with word size proportional to national weighted frequency. Across all three groups, the most frequently occurring terms were *shoot*, *gun*, and *hand/finger*, suggesting a predominance of firearm injuries involving handguns and extremity-related injuries. Additional common terms across groups included *accidental*, *thigh*, *handgun*, *leg*, *inflict*, and *cleaning*, reflecting recurrent themes related to accidental firearm discharge and handling activities.

Several group-specific patterns also emerged. Among NHW patients, terms such as *hunt*, *ricochet*, and *target* appeared more prominently, suggesting firearm use contexts associated with recreational or outdoor activities. Among NHB patients, *thigh*, *leg*, *accidental*, and *alcohol* were more frequent, indicating possible differences in injury patterns and situational contexts. Among Hispanic patients, terms including *head*, *inflict*, *alcohol*, and *cocaine* appeared more prominently, potentially reflecting variation in injury severity, mechanisms, or contextual circumstances surrounding injury events. These findings suggest that while broad patterns in self-inflicted firearm injuries are shared across groups, narrative text may capture meaningful differences in the circumstances and contexts surrounding these events.

**Fig. 3:**
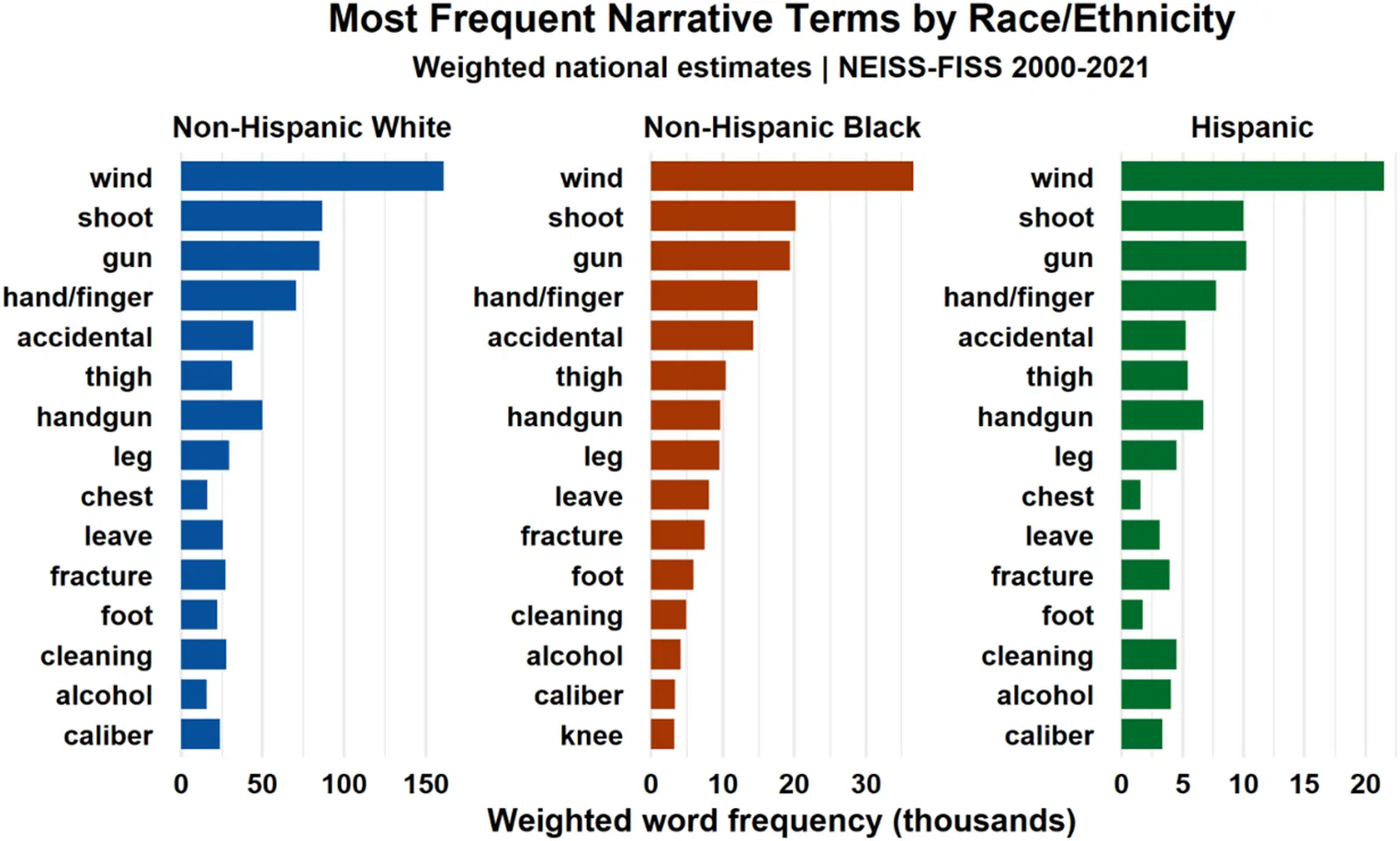
Most frequent narrative terms by race/ethnicity. Weighted national estimates. Top 15 terms by weighted frequency per group. NEISS-FISS 2000–2021

### Narrative Terms by Race/Ethnicity

**Fig. 4:**
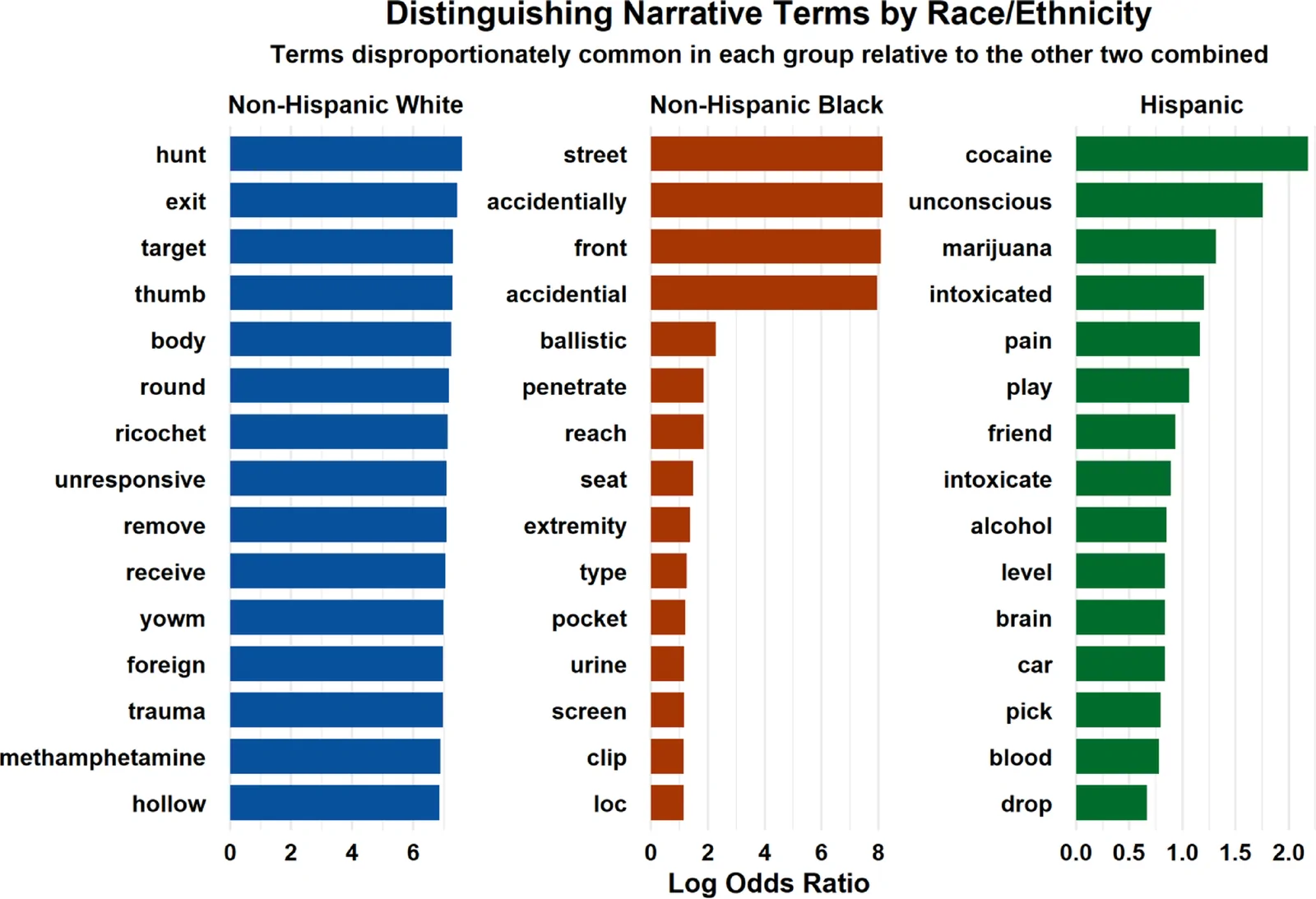
Distinguishing narrative terms by race/ethnicity (log odds ratio with Laplace smoothing, k = 0.5). Terms are disproportionately common in each group relative to the other two groups combined. Minimum 10 cases. NEISS-FISS 2000–2021

Figure 3 presents the most frequent terms by race/ethnicity. Figure 4 presents log-odds ratios that identify terms disproportionately represented within each racial and ethnic group relative to the remaining groups combined. Among NHW patients, distinctive terms included *hunt*, *ricochet*, and *target*, suggesting contexts related to hunting and recreational firearm use, as well as *thumb*, *body*, and *hollow*, which may reflect injury location and firearm or ammunition characteristics. The prominence of terms such as *hunt* and *target* is consistent with accidental firearm injuries occurring during hunting or shooting-related activities. Among NHB patients, distinguishing terms included *street*, *accidental*, *accidentally*, *front*, *seat*, and *ballistic*. These terms may reflect differences in incident location, injury circumstances, or mechanisms, with *street* and *seat* potentially indicating outdoor or vehicle-related contexts. The appearance of *accidental* and *accidentally* also aligns with a greater representation of narratives explicitly describing unintentional firearm discharge events. Among Hispanic patients, distinctive terms included *cocaine*, *unconscious*, *marijuana*, *intoxicated*, and *alcohol*. These findings are consistent with text-augmented analyses showing substantially higher levels of confirmed substance involvement among Hispanic patients after narrative-based recovery of information from structured fields. Collectively, these findings suggest that narrative text captures contextual features of firearm injury events that may not be evident in structured data alone and may reveal differences in the circumstances surrounding injuries across racial and ethnic groups.

### Intent from Narratives

**Fig. 5:**
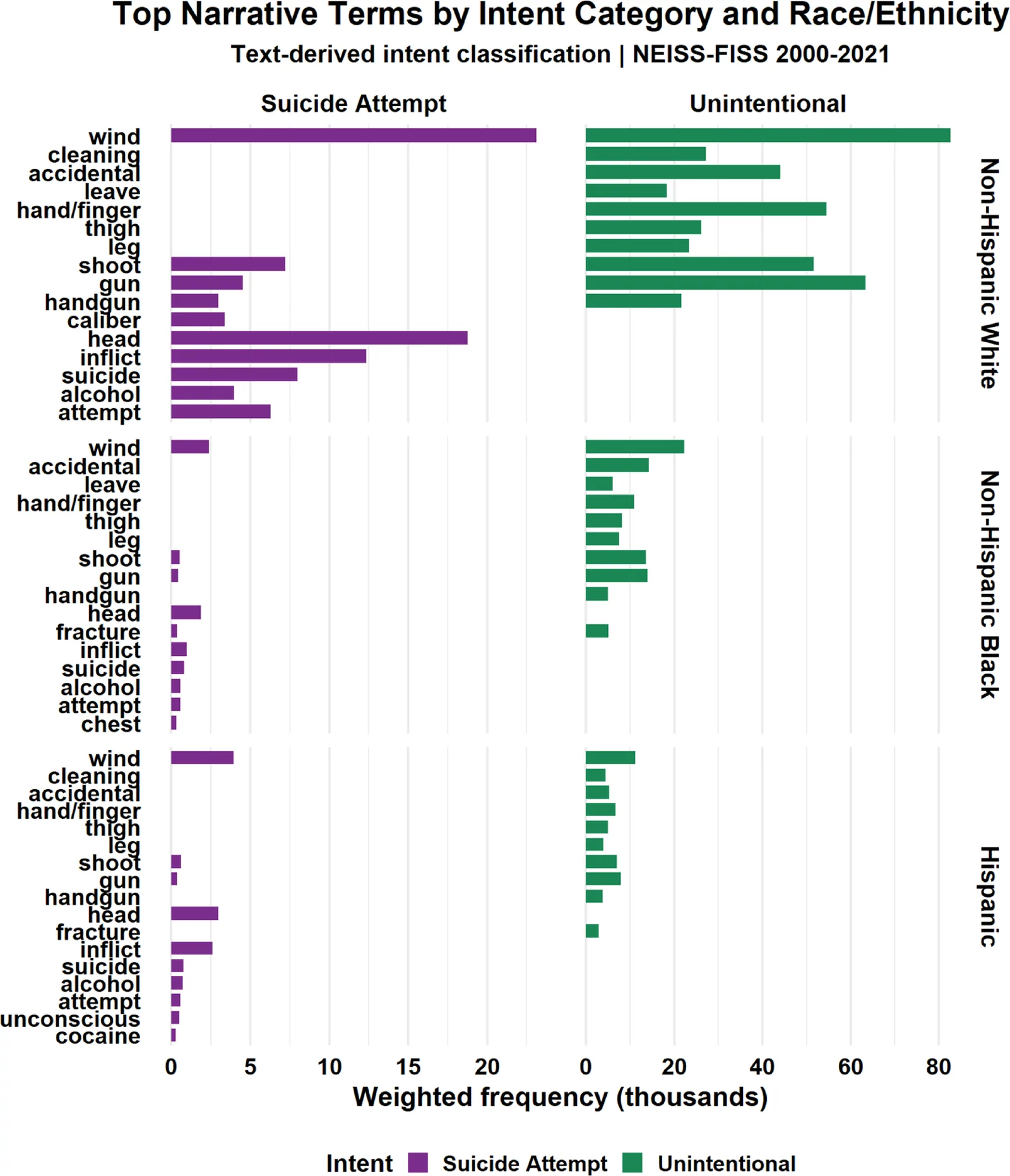
Top narrative terms by text-derived intent category (probable suicide attempt vs. probable unintentional discharge) and race/ethnicity. Purple = suicide attempt; green = unintentional. Top 10 weighted terms per group per category. NEISS-FISS 2000–2021

Figure 5 presents the top narrative terms stratified by text-derived intent category within each racial/ethnic group. Among probable suicide attempt narratives, *head* and *inflict* dominated across all groups, with *suicide* and *attempt* also prominent, consistent with ED coders explicitly documenting suicidal context. *Alcohol* appeared in suicide attempt narratives for all three groups. Among unintentional discharge narratives, *accidental*, *gun*, *hand/finger*, *cleaning*, *thigh*, and *leg* dominated across all groups, with *thigh* and *leg* particularly prominent in NHB patients.

### LASSO Prediction of Intent from Narrative Text

The temporally held-out elastic net classifier trained on cases from 2000 to 2010 (*n* = 1,420) and evaluated on cases from 2011 to 2021 (*n* = 2,345) demonstrated strong out-of-sample performance, achieving an accuracy of 97.1%, sensitivity of 98.0%, specificity of 92.2%, positive predictive value of 98.6%, negative predictive value of 88.9%, F1 score of 98.3%, and area under the receiver operating characteristic curve of 0.985. To reduce potential information leakage, terms directly involved in text-derived intent labeling and vocabulary from future observations were excluded during model development. Despite these restrictions, predictive performance remained strong, suggesting that broader contextual narrative patterns retained substantial discriminatory information.

**Fig. 6:**
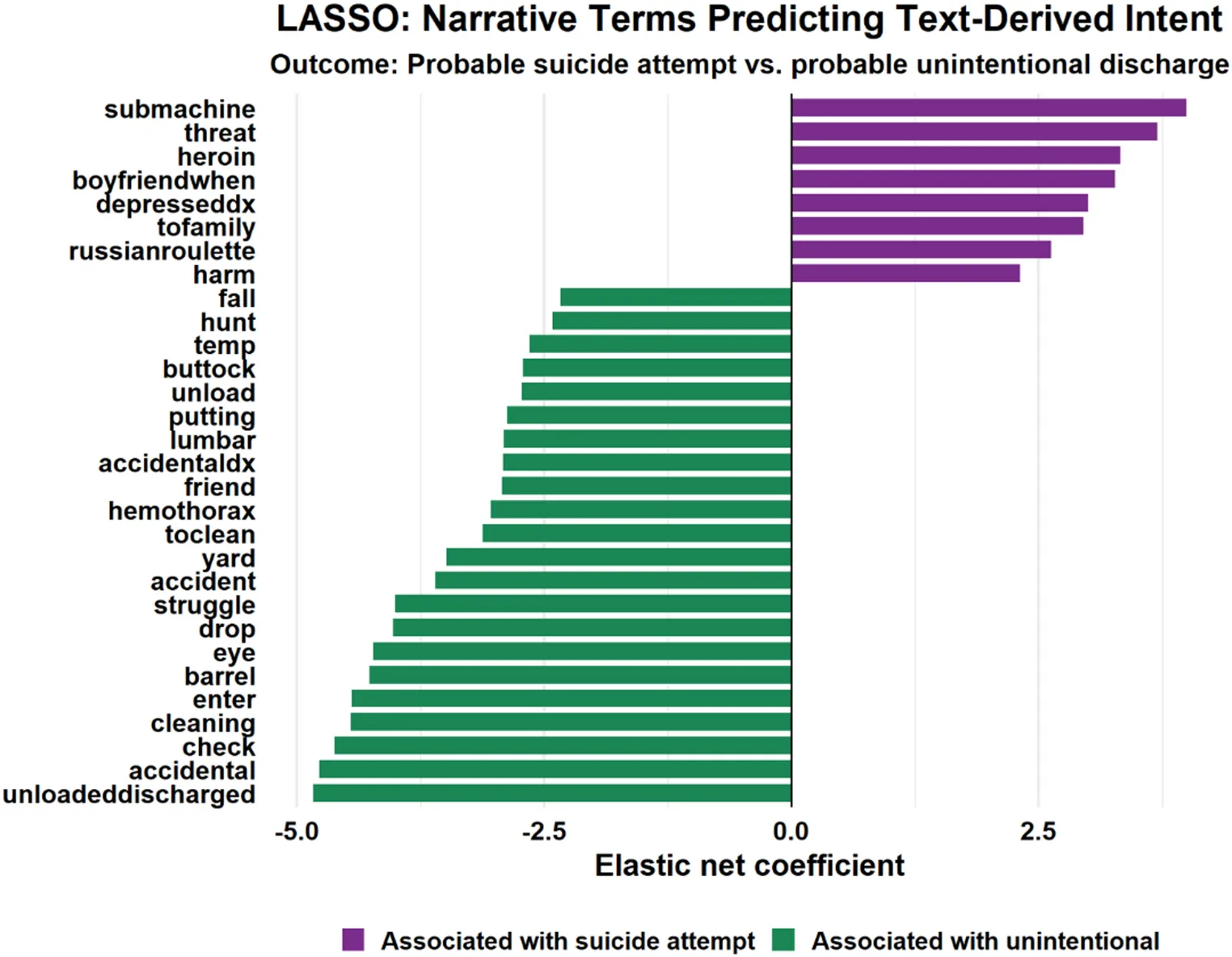
LASSO elastic net coefficients predicting text-derived intent of self-inflicted firearm injury. Outcome: probable suicide attempt (1) vs. probable unintentional discharge (0). Elastic net α = 0.8; 10-fold cross-validation within training period. Trained on 2000–2010 (*n* = 1,420); evaluated on held-out 2011–2021 (*n* = 2,345). Controls: age (standardized), sex, race/ethnicity, injury region. Top 30 narrative terms shown

Figure 6 presents the coefficient plot for the top retained narrative predictors. Terms associated with probable unintentional firearm discharge included combined bigrams, such as *unloadeddischarged*, *accidental*, *cleaning*, *barrel*, *drop*, *yard*, and *hunt*, reflecting firearm handling, maintenance, and accidental discharge contexts. In contrast, terms associated with probable suicide-attempt classifications included *threat*, *depresseddx*, *heroin*, *russianroulette*, *tofamily*, and *harm*, suggesting broader psychosocial and contextual patterns surrounding self-directed injury events. These findings indicate that narrative text retained meaningful predictive information even after the exclusion of explicit label-defining terminology.

## Discussion

Given the lethality of self-inflicted firearm injury, epidemiological surveillance in the United States has historically focused on fatal outcomes, which account for approximately 90% of self-inflicted firearm injuries [19]. However, a substantial number of individuals survive both accidental firearm injuries and suicide attempts, creating a growing need to better characterize the demographic trends and circumstances of nonfatal self-inflicted firearm injuries [7, 20]. This shift addresses an important gap in the literature because nonfatal firearm injuries impose substantial physical, psychological, and economic burdens on survivors and healthcare systems. Evaluating both fatal and nonfatal outcomes is therefore essential for informing clinical interventions and comprehensive prevention strategies [1, 19, 20].

### Racial and Ethnic Differences in Temporal Patterns and Epidemiology of Nonfatal Self-inflicted Firearm Injuries using Segmented Joinpoint Regression

Addressing Aim 1, the present study identified substantial racial and ethnic differences in temporal trends and injury patterns. Joinpoint analyses demonstrated that rates of nonfatal self-inflicted firearm injuries increased sharply during the mid-2010s, with NHB individuals experiencing the most pronounced increase. By 2021, NHB individuals exhibited markedly higher rates than either NHW and Hispanic populations. This pattern is supported by prior analyses of NEISS-FISS data documenting substantial increases in nonfatal firearm injuries among NHB individuals beginning around 2015 [2, 17]. Trauma registry studies similarly report that NHB patients are the only racial group exhibiting consistent increases in firearm-related hospitalizations over time [11]. Although these findings generally align with prior literature [21–23], they differ somewhat from Kaufmann [2], who reported that NHW individuals consistently exhibit the highest rates of intentional firearm self-harm, whereas NHB individuals experience the highest overall burden of firearm injury. This distinction appears important because our text-enhanced analyses demonstrated that approximately 65% of NHB individuals classified as “self-inflicted” experienced accidental firearm discharges rather than intentional suicide attempts. These findings suggest that apparent racial disparities may reflect differences in underlying injury mechanisms rather than differences in suicidal behavior alone.

The utility of narrative text to improve the classification of firearm injury intent.

Regarding Aim 2, a major finding was evidence suggesting systematic misclassification within structured administrative coding systems. Narrative analyses identified distinct contextual signatures across groups. Terms such as *hunt*, *ricochet*, and *target* among NHW individuals suggested recreational firearm environments, including hunting and shooting activities. Terms such as *street*, *seat*, and *accidental* among NHB patients suggested community, outdoor, or vehicle-related contexts. Hispanic patients showed distinctive substance-related terms, including *cocaine*, *marijuana*, *intoxicated*, *alcohol*, and *unconscious*, indicating more pronounced substance-use contexts and potentially greater injury severity. As well, we noted that 754 narratives contained explicit language consistent with suicidal self-harm but were classified as law-enforcement-related incidents. Within NEISS, law-enforcement injuries are intended to identify injuries caused by legal authorities during enforcement activities [24]; however, structured coding may instead capture contextual references to police presence rather than direct causation of injury.

These findings are highly consistent with prior literature documenting substantial limitations in structured administrative coding and medical records that frequently use stigmatizing language in documentation, which mirrors the same race-based inequities seen in medical outcomes and larger sociodemographic trends [25]. Studies such as [1, 15, 26] have shown that administrative systems frequently misclassify injury intent and often default uncertain firearm events to accidental categories. Kaufmann [27] similarly observed that coding errors can substantially distort estimates of firearm injury burden and case fatality. Collectively, these findings support growing evidence that structured coding alone may inadequately characterize the true intent and context of firearm injuries.

Our findings also align with emerging work using natural language processing and machine learning approaches to supplement structured surveillance systems. Parker [28] demonstrated that narrative text from NEISS-FISS can accurately recover missing contextual information, and [26] emphasized the value of machine learning methods for improving intent classification. Studies of law-enforcement-related firearm injuries similarly relied on manual narrative review, implicitly recognizing limitations of structured fields alone [3, 12].

### Racial and Ethnic Differences in Injury Mechanisms and Contextual Circumstances using Narrative Text Mining and Machine Learning Approaches, Distinguishing Probable Suicide Attempts from Unintentional Discharges

Finally, regarding our last aim, the present study demonstrated that the underlying mechanisms and circumstances of self-inflicted firearm injuries differ substantially across racial and ethnic groups. NHB patients predominantly experienced accidental firearm discharges involving lower-extremity injuries, whereas Hispanic and NHW patients demonstrated higher proportions of intentional suicide attempts characterized by craniofacial trauma and greater substance involvement.

These findings align with national epidemiological evidence showing that Black individuals experience the highest burden of nonfatal unintentional firearm injury [19]. Prior studies similarly report that accidental firearm discharges disproportionately involve extremity injuries [29], supporting our text-derived finding that many NHB self-inflicted cases involved lower-extremity injuries. Conversely, firearm suicide attempts disproportionately involve head and facial injuries because these injuries maximize lethality [7, 30]. Substance use findings among Hispanic patients also align with prior studies demonstrating frequent toxicological involvement among individuals with intentional firearm injuries [30]. Notably, narrative augmentation increased identified substance involvement among Hispanic patients by 67%, illustrating the value of supplementing structured fields with text-derived information.

This specific finding prompted an important analytical question: why did the analysis involving Hispanic individuals disproportionately highlight keywords associated with substance-related terminology? Although a definitive explanation lies beyond the scope of the present study, several factors may account for this pattern. Notably, these associations emerged within the narrative text analysis rather than within the initial structured coding categories. Potential explanations include inconsistencies in coding procedures, variations in self-reporting practices, clinician- or institutional-level documentation biases, and linguistic or cultural factors that influence how incidents are described and interpreted. Furthermore, differences in communication patterns, terminology, and culturally mediated expressions of distress or substance use may have contributed to the prominence of these terms in the narratives.

These findings suggest that prevention strategies should move beyond uniform approaches and instead incorporate context-specific interventions such as recreational firearm safety initiatives, urban firearm prevention strategies, and substance-integrated interventions. Specific narrative predictors identified by the elastic net model were also supported by prior literature. Rather than relying on explicit intent-defining terms, the final model identified contextual language associated with distinct injury circumstances. Terms associated with probable suicide attempts reflected broader psychosocial and behavioral contexts, whereas terms associated with probable unintentional firearm discharges reflected firearm handling and environmental settings. For example, terms related to firearm use and handling contexts aligned with prior evidence indicating that unintentional firearm injuries frequently involve accidental discharge events occurring during routine firearm activities or recreational contexts [29]. Importantly, the model retained strong predictive performance even after exclusion of terms directly tied to the outcome definition, suggesting that narrative text contains meaningful contextual information beyond structured administrative coding.

Overall, the findings suggest that machine learning approaches applied to unstructured narrative text offer a feasible and potentially accurate means of improving the identification of intent in firearm injuries. The present study’s LASSO classifier demonstrated that narrative language contains substantial predictive information beyond that captured by structured administrative codes. This finding is consistent with Parker [14], who demonstrated successful use of LASSO approaches in NEISS-FISS narrative data. Prior work by Barber [15] similarly emphasized that reliance on structured administrative coding alone introduces substantial classification error. Finally, these findings also suggest that integrating machine learning methods into firearm surveillance systems may improve public health monitoring and support more accurate identification of injury circumstances.

This study is not without limitations. One limitation is that the NEISS-FISS captures only ED presentations and misses self-inflicted injuries treated in outpatient settings or for which care was not sought. Intent classification from narrative text, while more informative than structured coding alone, remains prone to human error. For example, as discussed elsewhere [23]NEISS data is human-encoded from written and electronic ED records nationally, with varying levels of detail and different coding standards and standardization processes. Further, the dataset lacks key contextual characteristics (income, education, employment, insurance) and state identifiers, which may confound observed associations and warrant future inquiry. Moreover, we excluded some demographic groups due to small sample sizes, and our Hispanic sample size was relatively small; therefore, our results should be interpreted with some caution. Although a strict temporal train-test split was implemented to reduce information leakage and improve out-of-sample evaluation, the model was developed and tested within the same surveillance system. External validation using independent datasets remains necessary before clinical or surveillance implementation.

## Conclusions

Non-fatal self-inflicted firearm injuries among U.S. adults represent a heterogeneous injury population with substantial variation in mechanism and context across racial and ethnic groups. Findings suggest that many injuries classified as self-inflicted may involve unintentional firearm discharges rather than intentional self-harm, highlighting important limitations in current surveillance approaches. Emergency department narrative text substantially improved injury characterization by identifying otherwise unobserved information related to intent, substance involvement, and contextual circumstances. The predominance of unintentional mechanisms and the distinct racial and ethnic patterns observed suggest that prevention efforts should move beyond a single self-inflicted firearm injury framework and instead incorporate more tailored, context-specific strategies. Finally, the successful application of a LASSO classifier demonstrates the potential for machine learning and narrative text analysis to enhance firearm injury surveillance and improve characterization of firearm injury intent and context.

## Supplementary Information

Below is the link to the electronic supplementary material.

Supplementary Material 1

## References

[1] Miller, G. F., Barnett, S. B. L., Florence, C. S., McDavid Harrison, K., Dahlberg, L. L., & Mercy, J. A. (2024). Costs of Fatal and Nonfatal Firearm Injuries in the U.S., 2019 and 2020. *American Journal Of Preventive Medicine*, *66*, 195–204. 10.1016/j.amepre.2023.09.026 PMC10843794 38010238

[2] Kaufman, E. J., Song, J., Xiong, R., Seamon, M. J., & Delgado, M. K. (2024). Fatal and Nonfatal Firearm Injury Rates by Race and Ethnicity in the United States, 2019 to 2020. *Ann Intern Med United States*, *177*, 1157–1169. 10.7326/M23-2251 39074371

[3] Kaufman, E. J., Wiebe, D. J., Xiong, R. A., Morrison, C. N., Seamon, M. J., & Delgado, M. K. (2021). Epidemiologic trends in fatal and nonfatal firearm injuries in the US, 2009–2017. *JAMA Intern Med*, *181*, 237–244. 10.1001/jamainternmed.2020.6696 PMC7851729 33284327

[4] U.S. Government Accountability Office (2021). Firearm injuries: Health care service needs and costs [Internet]. Washington, DC: U.S. Government Accountability Office; June. Report No.: GAO-21-515. https://www.gao.gov/products/gao-21-515

[5] Orlas, C. P., Thomas, A., Herrera-Escobar, J. P., Price, M. A., Haider, A. H., & Bulger, E. M. (2021). Long-term Outcomes of Firearm Injury Survivors in the United States: The National Trauma Research Action Plan Scoping Review. *Annals Of Surgery*, *274*, 962. 10.1097/SLA.0000000000005204 34784664

[6] Geller, J. E., Teichman, A. L., Charles, E. J., Pierce, A., Patel, K., Park, J., et al. (2024). Firearm Injury, It’s Not Just Physical: The Adverse Impact on Patient-Reported Socioeconomic, Mental Health, and Quality-of-Life Outcomes. *American Surgeon*, *90*, 3038–3045. 10.1177/00031348241262434 38884300

[7] Rajasingh, C. M., Tennakoon, L., & Staudenmayer, K. L. (2018). Self-inflicted gunshot wounds: readmission patterns. *Journal Of Surgical Research*, *223*, 22–28. 10.1016/j.jss.2017.10.009 29433877

[8] CDC, & Fast Facts (2026). May : Firearm Injury and Death [Internet]. Firearm Inj. Death Prev. 2024 [cited 2026 May 22]. https://www.cdc.gov/firearm-violence/data-research/facts-stats/index.html. Accessed 22.

[9] Boeck, M. A., Strong, B., & Campbell, A. (2020). Disparities in Firearm Injury: Consequences of Structural Violence. *Curr Trauma Rep*, *6*, 10–22. 10.1007/s40719-020-00188-5

[10] Rowh, A. (2024). Emergency Medical Services Encounters for Firearm Injuries — 858 Counties, United States, January 2019–September 2023. MMWR Morb Mortal Wkly Rep [Internet]. [cited 2026 May 22];73. 10.15585/mmwr.mm7324a3 PMC11199023 38900705

[11] Salottolo, K., Sliter, R. J., Marshall, G., Palacio Lascano, C. H., Quan, G., Hamilton, D., et al. (2024). A joinpoint analysis examining trends in firearm injuries at six us trauma centers from 2016 to 2022. *Inj Epidemiol*, *11*, 18. 10.1186/s40621-024-00505-5 PMC11092259 38741167

[12] Loder, R. T., Young, A., & Atoa, B. (2021). Firearm injuries associated with law enforcement activity. *Journal Of Forensic And Legal Medicine*, *83*, 102249. 10.1016/j.jflm.2021.102249 34461598

[13] Holland, K. M., Rowh, A., & Zwald, M. L. (2025). Public health surveillance of nonfatal firearm injuries. Handb Gun Violence [Internet]. Elsevier; [cited 2026 May 22]. pp. 465–476. 10.1016/B978-0-323-95272-9.00018-8

[14] Parker, S. T., Barber, C., & Cook, P. J. (2025). Nonfatal Firearm Injury Surveillance in the US: An Update. Available SSRN 5208879 [Internet]. [cited 2026 May 22]; https://papers.ssrn.com/sol3/papers.cfm?abstract_id=5208879. Accessed 22 May 2026.

[15] Barber, C., Cook, P. J., & Parker, S. T. (2022). The emerging infrastructure of US firearms injury data. *Preventive Medicine*, *165*, 107129. 10.1016/j.ypmed.2022.107129 35803350

[16] United States Department of Health and Human Services, Centers for Disease Control and Prevention, National Center for Injury Prevention and Control (2023). Firearm injury surveillance study, 1993–2021 [Internet]. Ann Arbor, MI: Inter-university Consortium for Political and Social Research; 10.3886/ICPSR38923.v1.

[17] Bhagavathula, A., Price, J. H., & Khubchandani, J. (2025). Trends and Disparities in Non-fatal Firearm Injuries among Working-Age Adults in the United States, 2000–2021. *Journal Of Community Health*, *50*, 454–463. 10.1007/s10900-024-01431-9 PMC12069504 39702661

[18] Rinker, T. W. (2018). *textstem: Tools for stemming and lemmatizing text [Internet]*. Buffalo, New York;. http://github.com/trinker/textstem

[19] Kaufman, E. J., Song, J., Xiong, R., Seamon, M. J., & Delgado, M. K. (2024). Fatal and Nonfatal Firearm Injury Rates by Race and Ethnicity in the United States, 2019 to 2020. *Ann Intern Med American College of Physicians*, *177*, 1157–1169. 10.7326/M23-2251 39074371

[20] Quenzer, F., Givner, A., Dirks, R., Coyne, C. J., Ercoli, F., & Townsend, R. (2021). Self-Inflicted Gun Shot Wounds: A Retrospective, Observational Study of U.S. Trauma Centers. *West J Emerg Med*, *22*, 518–524. 10.5811/westjem.2021.4.49315 PMC8203010 34125021

[21] Watson-Grace, A. C., Barboza-Salerno, G. E., McCarthy, K. J. S., Harrington, T. R., Chang, Y., Stanek, C. J., et al. (2025). *A Descriptive Analysis of Suicide Trends by R/Ethnicity, Sex, and Age Categories in Santa Clara County, California from 2018–2023* (p. 100447). AJPM Focus. Elsevier. 10.1016/j.focus.2025.100447 PMC12856608 41624232

[22] Barboza-Salerno, G. E., Watson-Grace, A., Shockley-McCarthy, K., Harrington, T., Warren, K., & Steelesmith, D. (2025). The land cover paradox: Characteristics of blue-and green spaces within and beyond high-risk suicide clusters. *SSM-Popul Health Elsevier*, *31*, 101820. 10.1016/j.ssmph.2025.101820 PMC12169782 40530002

[23] Barboza-Salerno, G. E., Watson-Grace, A., Harrington, T., & Shockley McCarthy, K.. Sex-based differences in injury patterns and hospitalization from emergency department narratives of intimate partner violence, united states, 2013–2024. *Am. J. Emerg. Med.*, **103** (2025). 10.1016/j.ajem.2026.02.008 41720040

[24] Gotsch, K. E., Annest, J. L., Mercy, J. A., & Ryan, G. W. (2001). Surveillance for fatal and nonfatal firearm-related injuries—United States, 1993–1998. *MMWR Surveill Summ*, *50*, 1–32.

[25] Ivy, Z. K., Hwee, S., Kimball, B. C., Evans, M. D., Marka, N., Bendel, C., et al. (2025). Disparities in Documentation: Evidence of Race-Based Biases in the Electronic Medical Record. *J Racial Ethn Health Disparities*, *12*, 3294–3300. 10.1007/s40615-024-02132-8 PMC11836187 39160431

[26] Holland, K. M., Rowh, A., & Zwald, M. L. (2025). Public health surveillance of nonfatal firearm injuries. Handb Gun Violence [Internet]. Elsevier; [cited 2026 Apr 26]. pp. 465–76. 10.1016/B978-0-323-95272-9.00018-8

[27] Scantling, D., Orji, W., Hatchimonji, J., Kaufman, E., & Holena, D. (2021). Firearm violence, access to care, and gentrification: a moving target for American trauma systems. *Ann Surg LWW*, *274*, 209–217. 10.1097/SLA.0000000000004771 33605588

[28] Parker, W. J. (2020). The moral imperative of reproductive rights, health, and justice. *Best Pract Res Clin Obstet Gynaecol*, *62*, 3–10. 10.1016/j.bpobgyn.2019.07.006 31540808

[29] Tasigiorgos, S., Economopoulos, K. P., Winfield, R. D., & Sakran, J. V. (2015). Firearm injury in the United States: an overview of an evolving public health problem. *J Am Coll Surg LWW*, *221*, 1005–1014. 10.1016/j.jamcollsurg.2015.08.430 26422747

[30] Foote, C. W., Doan, X-L., Vanier, C., Cruz, B., Sarani, B., & Palacio, C. H. (2022). Suicide versus homicide firearm injury patterns on trauma systems in a study of the National Trauma Data Bank (NTDB). *Sci Rep Nature Publishing Group*, *12*, 15672. 10.1038/s41598-022-17280-2 PMC9485125 36123380

