# Supplementary material for: Racial and Ethnic Disparities in Non-Fatal Self-Inflicted Firearm Injuries Among Adults in the United States, 2000–2021
Source: J Community Health. 2026 Jun 17;51(5):770–84. doi: 10.1007/s10900-026-01592-9 (PMC13614987; doi:10.1007/s10900-026-01592-9)
Supplement: Supplementary file 1 [file 10900_2026_1592_MOESM1_ESM.docx]

**Appendix. Representative Emergency Department Narratives by Text-Derived Intent Category**

The following tables present 15 randomly sampled cases from each of the five text-derived intent categories. The Structured Intent column shows the original NEISS-FISS intent code as coded in the administrative record; the section heading shows the text-derived classification assigned by the narrative pattern-matching algorithm. The contrast between the Structured Intent column and the section heading for the Probable Suicide Attempt category illustrates the systematic coding discordance described in the Results and Discussion: all 15 randomly sampled cases in that section carry a structured code of Law Enforcement yet their narratives document unambiguous self-inflicted suicide attempts with no law enforcement involvement in the injury mechanism.

**Table 5. Probable Suicide Attempt (n = 15 randomly sampled cases)**

| **Year** | **Race/Ethnicity** | **Sex** | **Age** | **Structured Intent** | **Injury Region** | **Disposition** | **Narrative** |
| --- | --- | --- | --- | --- | --- | --- | --- |
| 2017 | Non-Hispanic White | Male | 38 | Law enforcement | Craniofacial/head-neck | Hospitalized | 38 YOM HAS SELF INFLICTED 9MM THROUGH & THROUGH GSW TO HEAD. DX: GSW TO HEAD W/EXTENSIVE SUPRATENTORIAL DAMAGE, HEMORRHAGIC SHOCK |
| 2011 | Other | Male | 64 | Law enforcement | Craniofacial/head-neck | Hospitalized | 64 YO M SELF INFLICTED GSW TO HEAD. DX GSW HEAD, CLOSED HEAD INJURY |
| 2006 | Non-Hispanic White | Male | 59 | Law enforcement | Craniofacial/head-neck | Hospitalized | PT SHOT SELF IN HEAD W/.22 CAL RIFLE. DX: CLOSED HEAD INJURY, GSW R FOREHEAD. |
| 2003 | Non-Hispanic White | Male | 34 | Law enforcement | Craniofacial/head-neck | Hospitalized | PT ATTEMPTED TO COMMIT SUICIDE WITH 22 RIFLE, SHOT SELF IN HEAD, MULTIPLE FOREIGN BODIES IN HEAD, ADMITTED TO SURGERY |
| 2008 | Non-Hispanic White | Male | 26 | Law enforcement | Craniofacial/head-neck | Transferred | PT PRESENTED TO ED UNRESPONSIVE W/ GSW TO TEMPLE AREA OF HEAD. USED 22 CALIBER HANDGUN AT CLOSE RANGE. SUICIDE ATTEMPT. |
| 2006 | Non-Hispanic White | Male | 54 | Law enforcement | Craniofacial/head-neck | Hospitalized | GSW TO HEAD. PT SHOT SELF IN HEAD. |
| 2019 | Non-Hispanic White | Male | 42 | Law enforcement | Craniofacial/head-neck | Hospitalized | 42 YOM GSW TO HEAD POSSIBLE SELF INFLICTED DX GSW TO HEAD |
| 2002 | Hispanic | Male | 26 | Law enforcement | Craniofacial/head-neck | Hospitalized | SELF INFLICTED GUN SHOT WOUND TO HEAD WITH PROFOUND BRAIN INJURY |
| 2005 | Non-Hispanic White | Male | 35 | Law enforcement | Craniofacial/head-neck | Hospitalized | GSW TO HEAD-SELF INFLICTED W/ .22 CALIBER GUN-LEFT SUICIDE NOTE-AT HOME-ETOH INVOLVED |
| 2011 | Non-Hispanic White | Male | 34 | Law enforcement | Craniofacial/head-neck | Hospitalized | GSW TO HEAD. 34-YEAR-OLD MALE SHOT SELF IN HEAD WITH A .32 CALIBER HAND GUN. |
| 2000 | Non-Hispanic White | Male | 28 | Law enforcement | Craniofacial/head-neck | Transferred | 28-YEAR-OLD MALE SHOT HIMSELF IN HEAD. SELF SUICIDE. DX: GUNSHOT WOUND TO HEAD. |
| 2012 | Non-Hispanic White | Male | 33 | Law enforcement | Craniofacial/head-neck | Hospitalized | GSW HEAD. 33-YEAR-OLD MALE SHOT SELF IN HEAD WITH A GUN. |
| 2018 | Hispanic | Male | 30 | Law enforcement | Trunk/internal | Hospitalized | 30-YEAR-OLD MALE WITH SELF INFLICTED GSW TO CHEST SUICIDE ATTEMPT DUE TO RELATIONSHIP ISSUES. DX HEMOTHORAX, GSW |
| 2006 | Other | Female | 41 | Law enforcement | Craniofacial/head-neck | Hospitalized | INTOXICATED IN SUICIDE ATTEMPT SHOT SELF FIRST IN RIGHT CHEEK, THEN LEFT CHEEK, BLOWING OUT ORBITS, CAROTID DISSECTION, SUBDURAL AND SUBARACHNOID HEMORRHAGE |
| 2019 | Non-Hispanic White | Male | 28 | Law enforcement | Craniofacial/head-neck | Hospitalized | 28-YEAR-OLD MALE WITH SELF INFLICTED GSW TO CHIN THROUGH LEFT FOREHEAD +ETOH BAL 152. DX: LEFT FRONTAL SDH, LAMINA PAPYRACEA FX, LEFT ORBITAL FLOOR FX |

*Note: All 15 cases above carry a structured intent code of Law Enforcement (value 3 in the NEISS-FISS intent field), yet every narrative describes a self-inflicted suicide attempt in a domestic or private setting with no law enforcement involvement in the injury mechanism. This illustrates the systematic coding discordance identified in this study (n = 754 total affected cases across 2000–2021).*

**Table 6. Probable Unintentional Discharge (n = 15 randomly sampled cases)**

| **Year** | **Race/Ethnicity** | **Sex** | **Age** | **Structured Intent** | **Injury Region** | **Disposition** | **Narrative** |
| --- | --- | --- | --- | --- | --- | --- | --- |
| 2016 | Other | Male | 57 | Self-harm | Multiple/all body | Hospitalized | 57-YEAR-OLD MALE POLICE OFFICER PLACING GUN INTO HOLSTER WHEN TRIGGER CAUGHT ON KEYS AND DISCHARGED INTO FINGER. LAC AND FX. |
| 2021 | Non-Hispanic Black | Male | 20 | Self-harm | Multiple/all body | Treated | 20-YEAR-OLD MALE WAS CLEANING HIS HANDGUN AND DID NOT KNOW THERE WAS A BULLET IN THE CHAMBER AND IT SHOT HIS LEFT LITTLE FINGER OFF. DX: AMPUTATION TO FINGER |
| 2015 | Non-Hispanic White | Male | 34 | Self-harm | Lower extremity | Hospitalized | 34-YEAR-OLD MALE WAS DRINKING ALCOHOL WHEN HE ACCIDENTALLY DROPPED HIS HANDGUN WHICH WENT OFF INJURING PATIENT. DX: TIBIA FX, ANKLE PAIN, ETOH INTOXICATION. |
| 2008 | Non-Hispanic White | Male | 38 | Self-harm | Lower extremity | Treated | ACCIDENTALLY SHOT SELF IN THE ANKLE AND HEEL. DX-GSW ANKLE, HEEL. |
| 2009 | Non-Hispanic White | Male | 27 | Self-harm | Upper extremity | Treated | GSW TO HAND. PT SHOT HIMSELF IN HAND WHEN CLEANING GUN |
| 2005 | Non-Hispanic Black | Male | 22 | Self-harm | Lower extremity | Treated | PATIENT ACCIDENTALLY SHOT SELF IN LEFT THIGH. DX-LACERATION LEFT THIGH GUNSHOT WOUND. |
| 2014 | Other | Male | 21 | Self-harm | Lower extremity | Treated | 21-YEAR-OLD MALE WAS CLEANING HIS GUN AND ACCIDENTALLY SHOT HIMSELF IN THE THIGH. DX GSW TO THIGH |
| 2021 | Non-Hispanic Black | Male | 24 | Self-harm | Lower extremity | Treated | 24-YEAR-OLD MALE HAD HANDGUN IN PANTS POCKET, ACCIDENTALLY SHOT SELF IN THE THIGH. DX: BALLISTIC WOUND THIGH |
| 2002 | Hispanic | Male | 61 | Self-harm | Upper extremity | Treated | PATIENT SHOWING OFF HIS ASSAULT RIFLE WHEN HE DROPPED IT AND IT WENT OFF AND SHOT SELF IN THE RIGHT LOWER ARM. PUNCTURE WOUND ARM |
| 2021 | Non-Hispanic Black | Male | 29 | Self-harm | Upper extremity | Treated | 29-YEAR-OLD MALE C/O GSW TO HAND. PATIENT WAS CLEANING HIS GUN AND ACCIDENTALLY SHOT HIS HAND. DX: GSW |
| 2013 | Non-Hispanic White | Male | 24 | Self-harm | Lower extremity | Hospitalized | 24-YEAR-OLD MALE WAS CLEANING HIS GUN WHEN IT DISCHARGED, SHOOTING HIS LEFT LOWER LEG. DX FRACTURE LT LOWER LEG ADMIT |
| 2007 | Non-Hispanic White | Male | 31 | Self-harm | Lower extremity | Treated | ACCIDENTAL GSW (SMALL CALIBER) TO FOOT. DX. GSW. |
| 2007 | Non-Hispanic Black | Male | 52 | Law enforcement | Lower extremity | Transferred | PATIENT WITH GUNSHOT WOUND TO FOOT, MEANT TO SHOOT SELF IN HEAD, GOT NERVOUS AND GUN WENT OFF INTO FOOT INSTEAD |
| 2007 | Other | Male | 23 | Self-harm | Multiple/all body | Hospitalized | DX LEFT MIDDLE FINGER THROUGH AND THROUGH: CHANGING CLOTHES, GUN FELL OUT OF HOLSTER AND HIT FLOOR, DISCHARGED AND HIT PATIENT'S FINGER |
| 2014 | Other | Male | 26 | Self-harm | Lower extremity | Treated | 26-YEAR-OLD MALE WITH LACERATION TO FOOT FROM GSW. STATES WAS CLEANING HIS GUN WHEN IT FIRED. REPORTS SMOKING MARIJUANA EARLIER. |

**Table 7. Self-Harm with No Text Signal (n = 15 randomly sampled cases)**

Cases in this category carry a structured self-harm intent code in the administrative record but contain no suicidal or accidental-mechanism language in the narrative, making them unclassifiable by text mining. Many appear clinically to be unintentional injuries where the structured code may be an artifact of the who_c = 1 (self-inflicted discharge) filter rather than a deliberate self-harm determination.

| **Year** | **Race/Ethnicity** | **Sex** | **Age** | **Structured Intent** | **Injury Region** | **Disposition** | **Narrative** |
| --- | --- | --- | --- | --- | --- | --- | --- |
| 2014 | Non-Hispanic White | Male | 36 | Self-harm | Lower extremity | Treated | 36-YEAR-OLD MALE COMPLAINING OF SELF INFLICTED GUNSHOT WOUND TO LEFT FOOT. DX GUNSHOT WOUND |
| 2008 | Non-Hispanic White | Male | 53 | Self-harm | Upper extremity | Treated | HAND INJURY FROM GUN SHOT WOUND TO LEFT HAND. DX-SELF INFLICTED GUN SHOT WOUND TO LEFT HAND, FRACTURE METACARPAL |
| 2015 | Non-Hispanic White | Male | 33 | Self-harm | Trunk/internal | Treated | 33-YEAR-OLD MALE WAS ATTEMPTING TO CLEAN GUN WITHOUT REMOVING AMMUNITION AND SHOT SELF IN TOE. DX LACERATION TOE |
| 2001 | Other | Male | 21 | Self-harm | Lower extremity | Treated | DX FOREIGN BODY RIGHT THIGH: TARGET PRACTICE WITH 12 GAUGE SHOT GUN, BULLET RICOCHETED OFF SOMETHING INTO LEG. |
| 2003 | Non-Hispanic White | Male | 19 | Self-harm | Upper extremity | Hospitalized | GSW TO LEFT SHOULDER. PATIENT WAS AT TARGET PRACTICE USING A 22 CALIBER HANDGUN AND WHEN FIRED IT RICOCHETED OFF AND STRUCK PATIENT IN SHOULDER. |
| 2008 | Hispanic | Male | 53 | Self-harm | Upper extremity | Treated | 53-YEAR-OLD MALE AT WORK USING SHOT GUN WITH SHOT BUT NO BULLETS; PIECE OF SHOT MAY HAVE GRAZED OR ENTERED PATIENT'S FOREARM. DX: PUNCTURE WOUND, F/B |
| 2000 | Non-Hispanic White | Male | 50 | Self-harm | Upper extremity | Hospitalized | PATIENT RECEIVED GSW TO SHOULDER. DX: SHOULDER PUNCTURE WOUND |
| 2018 | Non-Hispanic White | Male | 37 | Self-harm | Lower extremity | Treated | PATIENT WAS CLEARING THE CHAMBER OF THE 9MM AND IT FIRED HITTING HIS LEFT THIGH. GSW LEFT THIGH RETAINED BULLET FRAGMENT IN LEG |
| 2009 | Other | Male | 56 | Self-harm | Multiple/all body | Transferred | AMPUTATION FINGERTIP - 56-YEAR-OLD MALE PRESENTS WITH GSW - SHOT LEFT INDEX FINGERTIP OFF WITH AR15 WHEN SHOOTING AT HOME - FINGER NEAR BARREL TIP |
| 2014 | Non-Hispanic White | Male | 18 | Self-harm | Upper extremity | Treated | 18-YEAR-OLD MALE SUSTAINED A GUNSHOT WOUND TO HIS LEFT HAND WHILE COCKING THE PISTOL |
| 2004 | Other | Male | 33 | Self-harm | Lower extremity | Hospitalized | PATIENT ACCIDENTALLY SHOT LEG WITH HANDGUN. DX: GSW LEFT UPPER INNER LEG, ARTERIAL INJURY, COMPARTMENT SYNDROME |
| 2015 | Non-Hispanic White | Male | 43 | Self-harm | Multiple/all body | Treated | PATIENT WAS LOADING NEW AUTOMATIC PISTOL; CHAMBER CAME BACK AND CUT THUMB. LACERATION RIGHT THUMB |
| 2013 | Hispanic | Male | 23 | Self-harm | Upper extremity | Hospitalized | 23-YEAR-OLD MALE STATES HE WAS HOLDING FRIEND'S GUN AND IT FIRED. OPENING NOTED BETWEEN 2ND AND 3RD DIGITS. DX: GSW LEFT HAND. |
| 2010 | Non-Hispanic Black | Male | 22 | Self-harm | Upper extremity | Treated | 22-YEAR-OLD MALE ACCIDENTAL GSW TO HAND WITH A PISTOL |
| 2019 | Non-Hispanic White | Male | 22 | Self-harm | Lower extremity | Treated | 22-YEAR-OLD MALE SHOT HIMSELF IN THE THIGH WITH A GUN HE WAS USING TO SHOOT A PIG. DX: WOUND THIGH |

**Table 8. Law Enforcement Context (n = 15 randomly sampled cases)**

These 52 cases (0.7% of the race-stratified sample after reclassification) were classified as law enforcement context by the text algorithm because the narrative explicitly described police, officers, or law enforcement as directly involved in the incident circumstances — in contrast to the 754 reclassified cases above, whose narratives contain no such language.

| **Year** | **Race/Ethnicity** | **Sex** | **Age** | **Structured Intent** | **Injury Region** | **Disposition** | **Narrative** |
| --- | --- | --- | --- | --- | --- | --- | --- |
| 2021 | Non-Hispanic Black | Male | 30 | Law enforcement | Trunk/internal | Hospitalized | 30-YEAR-OLD MALE PRESENTS WITH SELF INFLICTED GSW TO ABDOMEN AND LEFT BUTTOCK. PER POLICE DEPARTMENT, PATIENT WAS BEING ARRESTED AND HAD A LONG STAND-OFF WITH POLICE. DX: GSW, CLOSED FX LT ILIAC WING. |
| 2015 | Non-Hispanic White | Male | 36 | Law enforcement | Craniofacial/head-neck | Hospitalized | 36-YEAR-OLD MALE WAS RUNNING FROM POLICE WHO CAUGHT UP TO HIM AND FOUND HE HAD SHOT HIMSELF TO HEAD. DX: GSW TO HEAD, SDH, OROPHARYNGEAL HEMORRHAGE, SHOCK |
| 2000 | Other | Male | 32 | Law enforcement | Craniofacial/head-neck | Transferred | GUN SHOT WOUND TO FACE - SELF INFLICTED - PATIENT FOUND BY POLICE IN HIS CAR - TRANSFERRED TO HOSPITAL |
| 2011 | Non-Hispanic White | Male | 53 | Law enforcement | Craniofacial/head-neck | Hospitalized | 53-YEAR-OLD MALE IN A STANDOFF WITH POLICE SO HE TOOK A HANDGUN AND SHOT SELF IN THE HEAD. SKULL FRACTURES |
| 2005 | Non-Hispanic White | Male | 42 | Self-harm | Multiple/all body | Treated | PATIENT: POLICE OFFICER, HAD FINGER GRAZED BY BULLET WHILE SHOOTING AT FIRING RANGE. DX: LEFT INDEX TIP AVULSION. |
| 2015 | Non-Hispanic White | Male | 40 | Self-harm | Upper extremity | Treated | 40-YEAR-OLD MALE INJURED UPPER ARM RAPIDLY FIRING SHOT GUNS DURING PRACTICE AS A POLICE OFFICER. DX- RIGHT UPPER ARM INJURY |
| 2009 | Non-Hispanic White | Male | 33 | Law enforcement | Craniofacial/head-neck | Hospitalized | PATIENT SHOT SELF TO FACE WITH .45 CALIBER AFTER BEING PULLED OVER BY POLICE. DX: GSW TO FACE WITH MASSIVE COMMINUTED FACIAL POLYFRACTURE. |
| 2011 | Hispanic | Male | 20 | Law enforcement | Lower extremity | Hospitalized | 20-YEAR-OLD MALE STATES HE WAS WALKING DOWN STREET WHEN HE HEARD GUNSHOTS AND FELT PAIN IN LEG. POLICE SUSPICIOUS OF SELF-INFLICTED. DX: GSW, THIGH. |
| 2003 | Non-Hispanic White | Male | 22 | Law enforcement | Craniofacial/head-neck | Hospitalized | GSW TO HEAD - SELF-INFLICTED WITH 9MM HANDGUN. POLICE CHASE AND RAN CAR INTO GUARDRAIL AND THEN SHOT HIMSELF. LATER DIED ON WARD. |
| 2020 | Non-Hispanic Black | Male | 57 | Law enforcement | Trunk/internal | Hospitalized | 57-YEAR-OLD MALE SELF INFLICTED GSW TO CHEST; ALSO SHOT AT POLICE OFFICER. UDS POSITIVE FOR BENZODIAZEPINES. DX GSW |
| 2021 | AI/AN | Female | 27 | Self-harm | Craniofacial/head-neck | Hospitalized | 27-YEAR-OLD FEMALE: POLICE RESPONDED TO PATIENT'S HOUSE FOR PERSON IN DISTRESS, PATIENT WAS HOLDING A GUN TO HER HEAD AND PULLED THE TRIGGER SHOOTING HERSELF IN HEAD. DX GSW HEAD SELF INFLICTED |
| 2021 | Non-Hispanic White | Male | 35 | Self-harm | Lower extremity | Treated | 35-YEAR-OLD MALE, INTOXICATED, REPORTS HAVING A GUNSHOT WOUND IN RIGHT THIGH FROM SEVERAL WEEKS AGO; WORSENING PAIN ABOVE RIGHT KNEE; PATIENT SHOT SELF - BROUGHT TO ED WITH POLICE (WON'T GIVE REASON WHY SHOT SELF) |
| 2010 | Other | Male | 22 | Self-harm | Upper extremity | Hospitalized | 22-YEAR-OLD MALE, POSSIBLE POLICE OFFICER, ACCIDENTALLY SHOT THROUGH HAND OVER SUSPECT BY 5TH FINGER. HAND OPEN FRACTURE |
| 2011 | Non-Hispanic White | Male | 24 | Law enforcement | Craniofacial/head-neck | Hospitalized | 24-YEAR-OLD MALE: PATIENT'S VEHICLE BEING CHASED BY POLICE CRASHED INTO GUARDRAIL. PATIENT GOT OUT OF CAR AND SHOT HIMSELF IN THE HEAD. DX GSW HEAD ADMIT |
| 2001 | Non-Hispanic Black | Male | 20 | Law enforcement | Craniofacial/head-neck | Transferred | PATIENT WAS INVOLVED IN SHOOTING LAST NIGHT, SHOT 3 BOYS, CALLED POLICE TO TURN SELF IN THEN SHOT SELF. DX: GUNSHOT WOUND TO HEAD. |

**Table 9. Indeterminate (n = 15 randomly sampled cases)**

Indeterminate cases contained no identifiable suicidal, accidental, or law enforcement language in the narrative, and were not coded as self-harm in the structured data. Many have sparse narratives with insufficient clinical detail to assign a text-derived intent classification.

| **Year** | **Race/Ethnicity** | **Sex** | **Age** | **Structured Intent** | **Injury Region** | **Disposition** | **Narrative** |
| --- | --- | --- | --- | --- | --- | --- | --- |
| 2009 | Non-Hispanic Black | Male | 40 | Law enforcement | Trunk/internal | Hospitalized | SELF INFLICTED GSW TO CHEST. GSW CHEST |
| 2005 | Other | Male | 49 | Law enforcement | Craniofacial/head-neck | Hospitalized | PATIENT SHOT SELF WITH A GUN INTO HEAD. DX: HEAD PUNCTURE WOUND/GSW |
| 2019 | Non-Hispanic Black | Male | 18 | Law enforcement | Craniofacial/head-neck | Hospitalized | 18-YEAR-OLD MALE WITH CERVICAL AND FACIAL INJURY AFTER SELF INFLICTED GSW TO MOUTH. DX: C3 PEDICLE FX; BILATERAL NASAL BONE FX |
| 2018 | Non-Hispanic White | Male | 43 | Law enforcement | Lower extremity | Hospitalized | 43-YEAR-OLD MALE PRESENTS WITH SELF INFLICTED GSW. PATIENT ATTEMPTING TO KILL SELF, INTENDED TO HIT FEMORAL ARTERY. DX: SUICIDAL IDEATION, GSW, RIGHT MID DISTAL TIBIA FX. |
| 2021 | Non-Hispanic Black | Female | 38 | Law enforcement | Upper extremity | Hospitalized | 38-YEAR-OLD FEMALE PRESENTS WITH A GSW TO THE RIGHT UPPER ARM. PER EMS, IT WAS SELF-INFLICTED. NO ADDITIONAL INFO PROVIDED ABOUT THE INCIDENT. DX: TRAUMA. |
| 2019 | Non-Hispanic White | Male | 23 | Law enforcement | Trunk/internal | Hospitalized | 23-YEAR-OLD MALE GSW TO CHEST AND FLANK IN SUICIDAL IDEATION ATTEMPT; ADMIT SICU. DX: HEMOTHORAX/PNEUMOTHORAX; GSW FLANK |
| 2010 | Non-Hispanic White | Male | 45 | Law enforcement | Lower extremity | Treated | 45-YEAR-OLD MALE WITH SELF INFLICTED GSW TO UPPER LEG. HAD BEEN DRINKING. |
| 2000 | Non-Hispanic White | Male | 62 | Unintentional | Craniofacial/head-neck | Treated | GUNSHOT WOUND TO HEAD. PATIENT SHOT HIMSELF IN THE HEAD. |
| 2019 | Hispanic | Female | 23 | Law enforcement | Trunk/internal | Hospitalized | 23-YEAR-OLD FEMALE HAS A SELF INFLICTED GSW TO CHEST. UNCONSCIOUS, UNRESPONSIVE. DX SELF INFLICTED GSW CHEST |
| 2000 | Non-Hispanic White | Female | 26 | Unintentional | Trunk/internal | Hospitalized | PATIENT RECEIVED GSW TO ABDOMEN. DX: PUNCTURE WOUND TO ABDOMEN |
| 2005 | Non-Hispanic White | Male | 60 | Law enforcement | Craniofacial/head-neck | Hospitalized | PATIENT WAS FOUND IN CHAIR AT HOME WITH GSW TO HEAD. DX: RIGHT SDH, FRONTAL HYPODENSITY, FRONTAL LOBE CONTUSION WITH HEMORRHAGE, MIDLINE SHIFT 10MM, FRACTURE |
| 2002 | Non-Hispanic White | Male | 37 | Law enforcement | Craniofacial/head-neck | Hospitalized | GSW TO HEAD WITH 38 CALIBRE, NO EXIT WOUND TO RIGHT TEMPORAL REGION, SELF-INFLICTED. DX: S/P GSW WITH RIGHT TEMPORAL BONE FX |
| 2010 | Other | Female | 54 | Law enforcement | Trunk/internal | Hospitalized | 54-YEAR-OLD FEMALE SELF INFLICTED GSW ABDOMEN +ETOH DUE TO STRESS AT HOME. DX GSW ABDOMEN |
| 2007 | Non-Hispanic White | Female | 58 | Unintentional | Craniofacial/head-neck | Treated | GSW HEAD |
| 2015 | Non-Hispanic White | Male | 40 | Law enforcement | Craniofacial/head-neck | Transferred | 40-YEAR-OLD MALE SELF-INFLICTED GUNSHOT WOUND TO CHIN/FACE INTUBATED. DX GUNSHOT WOUND FACE TRANSFERRED |

**Supplemental Figures**

**Fig. S1** *Injury region over time by race/ethnicity, 2000–2021. Weighted proportions by 5-year period.*

**Fig. S2** *Age group over time by race/ethnicity, 2000–2021.*

**Fig. S3** *Firearm type over time by race/ethnicity, 2000–2021.*

**Fig. S4** *ED disposition over time by race/ethnicity, 2000–2021.*

**Fig. S5** *Alcohol/substance involvement (structured coding) over time by race/ethnicity, 2000–2021.*

**Fig. S6** *Transported by EMS over time by race/ethnicity, 2000–2021.*
